# The impact of the development level of rural e-commerce on the depressive symptoms among rural older adult individuals

**DOI:** 10.3389/fpubh.2024.1477417

**Published:** 2024-12-11

**Authors:** Xiaofeng Xie, Siying Wei, Ling Zhu, Xiaoting Gan, Yong He, Rui Wang

**Affiliations:** ^1^West China Hospital, West China School of Nursing, Sichuan University, Chengdu, China; ^2^School of Economics, Sichuan University, Chengdu, Sichuan, China; ^3^School of Live Science and Engineering, Southwest University of Science and Technology, Mianyang, Sichuan, China; ^4^School of Marxism, Sichuan University, Chengdu, Sichuan, China

**Keywords:** e-commerce, depressive symptoms, rural older adult individuals, Difference-in-Differences, mediation analysis

## Abstract

**Background:**

In the context of China, where the demographic trend of population aging intertwines with the rapid advancement of information digitalization, rural older adult individuals, who are part of a vulnerable population, are witnessing a deteriorating depression status. The proliferation of rural e-commerce, which fuels the digital economic revolution in rural areas, is concurrently affecting the depressive symptoms among rural older adult individuals.

**Methods:**

This study uses longitudinal data from the China Health and Retirement Longitudinal Study (CHARLS) and applies a multi-period Difference-in-Differences (DID) model to explore how rural e-commerce affects the depressive symptoms among rural older adult individuals.

**Results:**

The findings indicate that the development of rural e-commerce can indeed improve the depressive symptoms among rural older adult individuals, with a particularly pronounced effect on those residing in the eastern and central regions of China, as well as those who do not live with their children. The mechanism analysis indicates that e-commerce improves depressive symptoms among rural older adult individuals by enhancing social interaction frequency and increasing financial support by children.

**Conclusion:**

Based on these insights, we recommend a targeted approach to implementing e-commerce policies in rural areas, focusing on innovative methods to improve the depressive symptoms among rural older adult individuals.

## 1 Introduction

Depression is one of the most common mental illnesses and a major cause of global disease burden (1).

The findings from Global Burden of Disease (GBD) study revealed that the worldwide age-standardized incidence rate of depressive disorders reached 3588.2 per 100,000 persons in 2019 (2). Due to the special physiological characteristics and social relations of the older adult, they often face a higher risk of depression. With the aging of the Chinese population, the depression problem of the older adult is becoming increasingly prominent (3). By the end of 2023, 297 million Chinese citizens are aged 60 and above, representing 21.1% of the total population (4). Notably, the overall prevalence rate of depression among the older adult is reported at 20.0% (5), with approximately 22.9% of older adult males and 30.6% of older adult females experiencing feelings of depression and other negative emotions (6). Compared to urban areas, rural older adult individuals confront more severe health challenges. Firstly, the aging population in rural areas is larger and more pronounced. According to data from the seventh national population census, the proportion of individuals aged 60 and above, as well as those aged 65 and above, in rural China is 23.81 and 17.72%, respectively, which are 7.99 and 6.61 percentage points higher than those in urban areas (7); Secondly, the rural older adult individuals face a higher risk of depression. In the face of accelerating urbanization, the mass migration of young and middle-aged rural labor forces has contributed to the depopulation of rural areas, leading to a notable rise in the phenomenon of “empty-nest” older adult populations (8). These rural older adult individuals often lack adequate daily care, financial support, and emotional comfort from their children (9), leading to a higher risk of depression in rural areas than those in urban areas. Furthermore, the economic underdevelopment, scarcity of medical resources, and lower health awareness among rural residents further exacerbate the situation, often resulting in the neglect of psychological issues among rural seniors.

The data from the “China Health and Retirement Report” indicate that the prevalence of depression among rural older adult individuals in China (38.3%) is significantly higher than in urban areas (22.2%) (10). Additionally, surveys have revealed that the incidence rate of depression among rural older adult individuals is 1.88 times that of urban areas (11). These findings suggest that the depressive symptoms among the older adult in rural China is a significant public health concern.

In an endeavor to meet farmers’ aspirations for a better life and improve their overall satisfaction, the Chinese government has given high priority to the development of rural e-commerce and has adopted a variety of policies to support its growth. In 2014, the Central Committee of the Communist Party of China officially recognized “rural e-commerce” as a key strategy for rural revitalization, providing a realistic theoretical foundation for the development of rural e-commerce (12). In 2023, the Central Committee of the Communist Party of China, in its No. 1 document, emphasizes the need to intensively implement the “Digital Commerce for Agriculture” and “Internet +” initiatives to facilitate the movement of agricultural products from rural to urban areas. In 2024, a joint document issued by the Ministry of Commerce and eight other departments, titled “Implementation Opinions on Promoting the High-Quality Development of Rural E-commerce,” highlights proactive measures for enhancing the high-quality development of rural e-commerce and establishes an improved ecosystem for the sector.

E-commerce, through the business model of selling goods and providing services on information platforms such as the Internet, breaks the traditional model of commercial activities mainly based on physical exchange or direct physical contact (13), realizes the informatization, electronization and networking of commercial and trade activities, boosts the development of the digital economy in rural areas and provides a new shopping experience for older adult individuals in these regions.

The growth of e-commerce in rural areas not only influences the daily lifestyles and digital consumption capabilities of older adults but also offers new perspectives for improving their mental health conditions, such as depression. However, limited research addresses the relationship between rural e-commerce development and depression among rural older adult individuals. This study, therefore, focuses on depressive symptoms among rural older adult individuals and examines the role of e-commerce development in addressing it, aiming to provide a theoretical foundation for improving depressive symptoms among rural older adult individuals.

## 2 Literature review

### 2.1 Current status of depressive symptoms among rural older adult individuals

Depression is a serious mental health condition, primarily manifested by persistent sadness, diminished interest, difficulty concentrating, and lack of energy (14). It typically leads to a decline in quality of life, increased mortality, and higher risks of suicide, contributing to a substantial societal burden (15). Due to the unique physiological characteristics, economic status, and social relationships of rural older adult individuals, they often face a higher risk of depression (16). A meta-analysis reveals that the prevalence of depressive symptoms among older adult individuals in rural China reaches 24.0% (5). Another study investigating differences in depressive symptoms between urban and rural older adult populations shows that the incidence of depressive symptoms among rural older adult (12.41%) is higher than that of urban older adult (10.13%) (17). These findings, consistent with other research, confirm a significant urban–rural disparity in the incidence of depression among the older adult in China (18), underscoring the urgent need for targeted attention and intervention to address depression in rural older adult individuals.

### 2.2 Factors influencing the depressive symptoms among rural older adult individuals

The depressive symptoms among rural older adult individuals are intricately linked to a variety of factors, including their individual characteristics, health related risk factors, family support, and social environment. Primarily, in terms of individual characteristics of older adult individuals, the depressive symptoms status of rural seniors varies according to gender, educational background, and health status. Scholars have observed gender differences in depressive symptoms among Chinese rural older adult individuals, attributing the phenomenon to women’s tendency to be more sensitive and nuanced, and thus more susceptible to psychological problems, than men (19); Maier’s research shows that as educational attainment increases, the prevalence of depression among older adult individuals gradually decreases (20); Secondly, in terms of mental health-related risk factors for older adult individuals, older adult individuals with better self-rated health and healthier lifestyles had a lower risk of depression (21), and older adult individuals who slept less than 6 hours were more likely to experience depressive symptoms (22); In a longitudinal study, Liu et al. have found that the prevalence of depression among older adult individuals with chronic conditions was 1.17 times higher than among those without chronic conditions (23), and even higher among those with more than two to three chronic conditions (24). Furthermore, based on the millennia-old tradition of filial piety, the support of children is an important pillar for the survival of the older adult in rural China and plays an important role in their mental health (25). Research shows that the economic support, emotional interaction, and care giving behaviors of children can reduce negative emotions in older adult individuals (26), improve their subjective well-being (27), and enhance their autonomy in later life. Living with children is beneficial to the depressive symptoms among rural older adult individuals (28). However, in the process of modernization, a significant number of young and middle-aged rural workers are choosing to migrate to urban areas for work and business (29), increasing the physical distance between generations (30). This has made traditional care models impractical (31), resulting in varying degrees of deterioration in the physical and mental health of older adult individuals (32). In addition, the social environment also influences the psychological well-being of older adult individuals. A longitudinal analysis conducted in Europe suggests that inadequate social networks and levels of participation are associated with depressive symptoms among older adult individuals (33). Engaging in social activities has been shown to improve the depressive symptoms among rural older adult individuals (34); Xue et al. also found that older adult individuals with higher socioeconomic status are less likely to experience negative emotions such as loss and depression (35). Furthermore, researchers have indicated that good access to healthcare services and higher family income are beneficial for alleviating depressive symptoms among rural older adult individuals.

### 2.3 E-commerce and the depressive symptoms among rural older adult individuals

Under the guidance of China’s National E-Commerce Demonstration Cities (NEDC) initiative and the significant promotion of digital platforms and internet infrastructure (36), e-commerce rapidly emerges as one of the fastest-growing sectors. E-commerce is defined as “using electronic information technology to conduct business between trading partners, using or not using electronic data interchange (EDI), using or not using the Internet” (37), unlike traditional social media and internet technologies, e-commerce places greater emphasis on the online process of goods trade, including product selection, consultation, and sharing of shopping experiences. The widespread adoption of online sales models expands consumer channels, creates diverse employment opportunities, and significantly boosts economic growth (38). According to the statistics, the online retail sales in China reached 13.1 trillion yuan in 2021, of which online retail sales of physical goods accounted for over 82%, accounting for 24.5% of the total retail sales of consumer goods (39). In addition to economic benefits, e-commerce influences residents’ shopping behaviors and enriches their participation in online social interactions. Furthermore, the optimization and upgrading of logistics systems generate new services, such as offline parcel collection and returns, which, to some extent, promote residents social interactions and subsequently impact their mental health. Studies indicate that the digital economy, primarily measured by e-commerce, expands social interaction opportunities and reduces depression rates among middle-aged and older adult individuals (40). However, among university students, irrational online shopping and shopping addiction associated with e-commerce may increase the risk of depression (41). These findings suggest that the impact of e-commerce on residents’ depressive symptoms warrants further investigation.

Current studies mostly focus on the consumption capacity of residents and its income-increasing effect on rural areas under the development of e-commerce economy (42), paying little attention to exploring the impact of e-commerce on the depressive symptoms of residents and its mechanism of action, especially for the rural older adult who are socially marginalized. In view of this, this study takes the depressive symptoms among rural older adult individuals in China as the main research object to explore new dimensions of the relationship between e-commerce and depressive symptoms that have received less attention in the previous literature, with the expectation of providing references for improving the depressive symptoms among rural older adult individuals.

### 2.4 Theoretical foundation and research hypotheses

The theoretical foundation of this study is primarily based on Role Theory, Activity Theory, and Family Support Hypothesis. These theories provide important perspectives for understanding how rural e-commerce affects depressive symptoms among rural older adult individuals, particularly in the context of social interaction and financial support by children.

Role Theory was initially developed to explore how individual behavior changes due to variations in environment and identity (43). As research has progressed, the conceptual framework of this theory has become increasingly comprehensive, providing a theoretical foundation for understanding the role transformations of older adult individuals in the context of e-commerce in rural areas. During the aging process, rural older adult individuals undergo two primary role transformations (44). Firstly, as physical abilities decline and learning capacities diminish, they transition from active work roles to leisure roles, with many forced to withdraw from work and opt for home-based retirement (45). This shift diminishes their social functionality and contracts their social networks, resulting in a significant reduction in opportunities for social interaction. The abundance of leisure time, coupled with the lack of recreational facilities in rural areas, leaves many older adults feeling directionless, further exacerbating feelings of loneliness, loss, and confusion about the meaning of life. Secondly, cognitive and physical limitations compel rural older adult individuals to gradually cede their dominant roles within the family to their adult children, and depend on them for care and financial support (46). If the older adult fail to adapt to this transition, they may experience a profound sense of loss, which can trigger negative emotions such as anger and depression, ultimately affecting family harmony.

In this context, the development of rural e-commerce can effectively alleviate depressive symptoms among older adult individuals in two key ways. Firstly, the online trading activities facilitated by e-commerce create novel and purposeful forms of leisure for rural seniors, enabling them to easily browse and purchase a variety of goods that meet both their basic needs and emotional desires. This sense of engagement enhances their autonomy and satisfaction, helping them to redefine their life roles and alleviate the negative emotions stemming from the contraction of social relationships. Secondly, e-commerce also facilitates interaction between rural older adult individuals and their adult children. Through e-commerce platforms, children can not only guide their parents on how to shop online but also directly purchase necessary items for them. This interaction allows seniors to feel valued and cared for by their family members, alleviating feelings of role displacement while fostering joy and a sense of well-being, ultimately enhancing their mental health.

Activity Theory asserts that participation in social interaction is essential for the mental health of older adult individuals (47). We now live in an information age where the concept of social interaction has been significantly expanded. In addition to traditional face-to-face communications, rural seniors can engage in online social interactions, such as those facilitated by e-commerce, to connect with people around the world (48).

E-commerce platforms offer various avenues for interaction with merchants, other buyers, and live stream hosts, including product consultations, shopping shares, and live selling sessions (49, 50). This not only alleviates the social participation inequalities faced by older adult individuals in urban and rural areas due to inadequate infrastructure and service accessibility, but also expands their social circles and effectively reduces feelings of social isolation and loneliness (51). Additionally, the offline processes associated with e-commerce, such as product pickup and returns, facilitate communication between rural older adult individuals and delivery personnel. Therefore, e-commerce promotes the social interaction frequency among rural seniors from both online and offline perspectives. Based on this, the present study selects the social interaction frequency among rural older adult individuals as a mediating variable to explore the relationship between e-commerce and depression symptoms in this population.

According to the Family Support Hypothesis, emotional and financial support from family members can effectively reduce depression levels among the older adult (52). Multiple studies have shown that financial support by children has a positive impact on the mental health of seniors (53, 54). Rooted in the traditional Chinese concept of filial piety, such financial assistance not only provides material help but also signifies respect and care for parents (38). This emotional support can significantly enhance the life satisfaction of older adult individuals.

The development of e-commerce provides new avenues for family support. On one hand, children can directly meet their parents’ online shopping needs through financial transfers. On the other hand, they can actively purchase necessary items for their parents. These actions not only satisfy the material needs of older adult individuals but also enhance their perception of family support. Through these means, rural seniors can feel valued, which, in turn, boosts their sense of happiness and promotes their mental well-being.

Based on the preceding discussion, the following hypotheses are proposed:

*Hypothesis 1:* The development of e-commerce has a significant impact on the depressive symptoms among rural older adult individuals.

*Hypothesis 2:* The development of e-commerce affects the depressive symptoms among rural older adult individuals by increasing their social interaction frequency.

*Hypothesis 3:* The development of e-commerce affects the depressive symptoms among rural older adult individuals by increasing the financial support by children.

**Figure 1:**
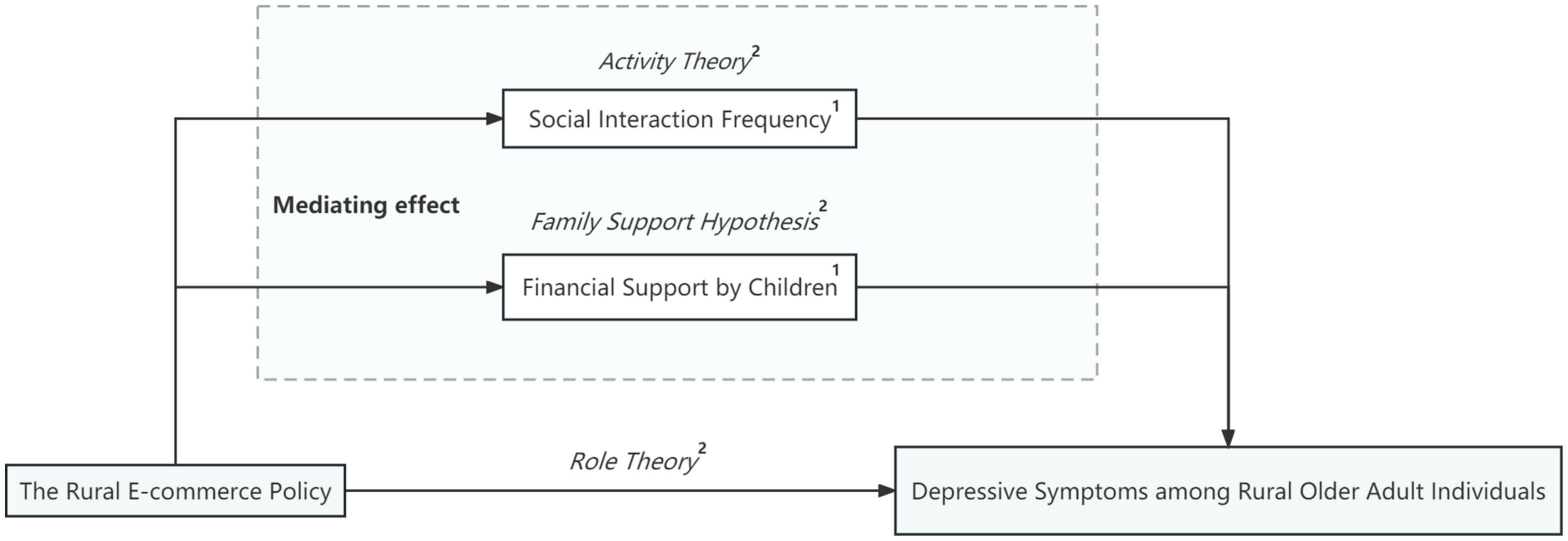
Conceptual framework. 1: The mediating variable; 2: Theoretical foundation.

The Conceptual Framework of this study is illustrated in Figure 1. This figure indicates that the development of rural e-commerce can directly and indirectly improve depressive symptoms among rural older adult individuals. First, based on “Role Theory” rural e-commerce facilitates role adaptation in the older adult, directly impacting their depressive symptoms. Second, supported by “Activity Theory,” which emphasizes that “participation in social activities is crucial for the mental health of older adult individuals,” the study finds that the growth of rural e-commerce enhances the social interactions frequency among rural older adult individuals, thereby alleviating their depressive symptoms. Additionally, the mechanism by which rural e-commerce development improves depressive symptoms through increased financial support by children is supported by the “Family Support Hypothesis.”

## 3 Empirical research design

### 3.1 Data source

The data underlying this study come from two sources. The first is the China Health and Retirement Longitudinal Study (CHARLS) database, a large interdisciplinary survey project hosted by the National Institute of Aging at Peking University and conducted by the Chinese Academy of Social Sciences. The survey is randomized and has been collecting baseline data on a variety of aspects, including basic information, health status, and health care, from families and individuals aged 45 and older across China since 2011. Subsequent follow-up surveys were conducted in 2013, 2015, 2018, and 2020. To date, the CHARLS database has been used to survey 19,000 respondents from 12,400 households in 450 communities (villages) across 150 counties in 28 provinces (autonomous regions and municipalities) nationwide. This study uses five datasets from the CHARLS database, from its inception in 2011 to 2020, for empirical analysis. The second source is the China Regional Statistical Yearbook, an annual publication compiled by the National Bureau of Statistics that comprehensively reflects the economic and social development of various regions within China. The data on city-level control variables included in this study are extracted from this document.

### 3.2 Variable construction

#### 3.2.1 Dependent variable

In the CHARLS database, the depression status of middle-aged and older adult participants is primarily assessed using the CES-D-10 scale (55). This scale has a high Cronbach’s alpha coefficient of 0.799 and a KMO value of 0.889, indicating robust psychometric properties. It consists of eight items focusing on positive emotions and two items focusing on negative emotions, as detailed in Table 1. Each item is rated on a four-point scale reflecting the frequency of the described emotions. The scale’s response options are calibrated to adjust scores based on the polarity of the statements, culminating in a CES-D score. A higher score indicates higher levels of depression.

**Table 1:** Indexes of depressive symptoms status.

No	Question
1	I was bothered by things that do not usually bother me
2	I had trouble keeping my mind on what I was doing
3	I felt depressed
4	I felt everything I did was an effort
5	I felt hopeful about the future
6	I felt fearful
7	My sleep was restless
8	I was happy
9	I felt lonely
10	I could not get “going”

#### 3.2.2 Core explanatory variables

In 2014, the pilot program for e-commerce in rural areas was officially launched. Since the establishment of an e-commerce platform requires a certain amount of time, this study sets the formal intervention time of the rural e-commerce policy at 2015. The level of development of rural e-commerce is taken as the core explanatory variable, and its effect on the depressive state of rural older adult is examined. The dummy variable for areas where the rural e-commerce policy has been implemented is set to 1, while for those where it has not been implemented, it is set to 0 (56).

#### 3.2.3 Control variables

In order to accurately capture the effect of e-commerce development level on depressive symptoms among rural older adult individuals and reduce endogeneity, it is necessary to introduce some control variables. The depressive symptoms among rural older adult individuals are influenced by various factors, which are examined in this study from two perspectives: individual characteristics and the social environment. First, regarding individual characteristics, it is observed that older adult individuals are more prone to severe depressive symptoms (57). Women are particularly vulnerable to negative emotions, leading to conditions such as loneliness and depression (58). Besides, seniors who are married or have higher levels of education tend to have better mental health (59). In contrast, individuals who smoke, consume alcohol excessively, and experience poor health have a higher risk of developing mental disorders (60–62). In addition, from the perspective of the social environment, rural areas generally suffer from inferior economic conditions and healthcare services. Rural older adult individuals are more likely to be exposed to risk factors, such as social exclusion, while having limited access to protective factors, such as education and specialized medical care (63). This situation makes them more susceptible to mental health disorders. Furthermore, research has observed that lower population densities often correlate with an increased risk of depressive symptoms issues (64).

In summary, based on the factors influencing the depressive symptoms among rural older adult individuals and with reference to the study by Nie et al. (65), this study selects individual characteristic variables and social environment variables as control variables. The individual characteristic variables of the older adult include age, self-rated health (SRH), education level (Education), marital status (Marriage), smoking status (Smoke), drinking status (Drink), and gender. The family support and social environment variables include the logarithm of income (Lnincome), the logarithm of GDP growth rate (LnGDP), the logarithm of GDP *per capita* (LnmGDP), the logarithm of the number of persons per million square kilometers (Lnmidu), agricultural structure (AS), and the logarithm of the number of practicing physicians per 10,000 persons (Lnphysicians). For the purpose of avoiding bias such as heteroskedasticity due to excessive numerical difference, some of the control variables in this paper have been processed logarithmically. The main variables of this study are shown in Table 2.

**Table 2:** Variable summary table.

Variable type	Variable name		Measurement standard	Unit
Dependent variable	Depression level		CES-D-10 scale	–
Independent variable	DID		0 = Policy not implemented in the sample area in the current year; 1 = Policy implemented in the sample area in the current year	–
Control variables	Personal characteristics	Age	Age during the survey year	Years
		Self-rated health (SRH)	1 = Very good; 2 = Good; 3 = Fair; 4 = Poor; 5 = Very poor	–
		Education	1 = Below primary school; 2 = Primary school; 3 = Middle school; 4 = High school and above	–
		Marriage	1 = Married and living with spouse; 2 = Temporarily living apart due to work, etc. 3 = Separated, no longer living with spouse; 4 = Divorced; 5 = Widowed; 6 = Never married	–
		Smoke	0 = Non-smoker; 1 = Smoker	–
		Drink	0 = Non-drinker; 1 = Drinker	–
		Gender	1 = Male; 2 = Female	–
	Social environment	Lnincome	Logarithm of income	–
		LnGDP	Logarithm of GDP growth rate	–
		LnmGDP	Logarithm of *per capita* GDP	–
		Lnmidu	Number of people per million square kilometers	People
		Agricultural structure (As)	Proportion of agriculture in total primary industry output	%
		Lnphysicians	Proportion of agriculture in total primary industry output	–

### 3.3 Empirical model

To promote rural economic development and reduce the urban–rural economic gap, China has gradually implemented the E-commerce to Villages plan. In 2014, China launched the first batch of pilot programs for the policy, with 56 regions receiving support to establish rural e-commerce demonstration counties. The policy aims to improve e-commerce infrastructure, enhance service levels, provide e-commerce training, and cultivate e-commerce talent in the pilot areas. Subsequently, the second and third batches of pilot programs were launched, gradually expanding the scope and scale of the initiative (66).

The Difference-in-Differences (DID) method can be used to assess the impact of policies by comparing changes in the policy intervention group and the control group before and after the policy intervention, and it is currently widely applied in the field of policy evaluation (9). It is typically used to assess the effectiveness of policies implemented during the same period. However, the rural e-commerce policy involves different pilot regions and varying implementation timelines, providing a quasi-natural experimental environment for this study. Therefore, a staggered Difference-in-Differences model is employed for regression analysis. In this model, regions that implemented the rural e-commerce policy are considered the treatment group, while regions that did not implement the policy serve as the control group. To explore the impact of e-commerce development levels on the depressive status of the rural older adult, The established model is as follows:


$$\mathrm{Depressio}n_{\mathrm{it}}=\alpha_{0}+\alpha_{1}DID_{\mathrm{it}}+\sum \alpha_{2}\mathrm{Contro}l_{\mathrm{it}}+\gamma_{i}+\mu_{t}+\varepsilon_{\mathrm{it}}$$

In this model, $\mathrm{Depressio}n_{\mathrm{it}}$ represents the depressive symptoms among rural older adult individuals. The DID (Difference-in-Differences) serves as the core explanatory variable. If the rural area where the older adult individual resides implements the e-commerce policy at time t, the DID takes a value of 1; otherwise, it is 0. This variable acts as the interaction term in traditional Difference-in-Differences approaches. $\sum \alpha_{2}\mathrm{Contro}l_{\mathrm{it}}$ represents a series of control variables for individual characteristics and social environments among rural older adult individuals, $\gamma_{i}$ is regional fixed effects, and $\mu_{t}$ is annual fixed effects.

## 4 Empirical results and analysis

### 4.1 Descriptive statistics of major variables

Table 3 presents the descriptive statistics of the main variables in this study. A total of 10,134 rural older adult individuals participated in the survey, with a median age of 68 years (ranging from 60 to 98). The average score on the CES-D-10 depression scale is 8.639 ± 5.692.

**Table 3:** Descriptive statistics for each of the main variables.

Variables	Obs	Mean	S. D.	Min	Max
Depression	10,134	8.639	5.692	0.000	30.000
DID	10,134	0.613	0.487	0.000	1.000
Age	10,134	68.173	5.966	60.00	98.000
Srh	10,134	2.932	1.001	1.000	5.000
Education	10,134	1.643	0.889	1.000	4.000
Marriage	10,134	0.840	0.366	0.000	1.000
Smoke	10,134	0.277	0.448	0.000	1.000
Drink	10,134	0.337	0.473	0.000	1.000
Lnincome	10,134	8.944	0.908	2.485	12.886
Lngdp	10,134	2.091	0.486	−1.470	2.884
Lnmgdp	10,134	10.436	0.544	8.842	12.201
AS	10,134	−1.832	0.501	−4.214	−0.677
Lnmidu	10,134	489.975	265.917	9.440	1356.570
Lnphysicians	10,134	21.814	4.344	12.30	33.250

### 4.2 Baseline regression results

This study investigates the impact of the development level of rural e-commerce on depressive symptoms among rural older adult individuals, with the basic regression results presented in Table 4. Column (1) of Table 4 provides the estimation results without any control variables, where the DID coefficient is significantly negative at the 1% level, indicating that the development of rural e-commerce has improved the depressive symptoms among rural older adult individuals. In Column (2), individual characteristics and social variables of the older adult are added, including age, health score, educational level, marital status, smoking and drinking habits, gender, logarithm of income, logarithm of GDP growth rate, logarithm of *per capita* GDP, the proportion of agriculture in the total output of the primary industry, and the logarithm of the number of people per million square kilometers, as well as the logarithm of the number of practicing assistant physicians per 10,000 people. The regression results show that although the DID coefficient remains negatively significant at the 1% level, the model’s goodness of fit increases, further demonstrating the robustness of the baseline regression.

**Table 4:** Benchmark regression of the effect of e-commerce development level on depressive symptoms among rural older adult individuals.

	Depression (1)	Depression (2)
DID	−1.4115^***^	−1.4995^***^
	(0.2192)	(0.2341)
Age		−0.0629
		(0.1069)
Srh		−0.6426^***^
		(0.0723)
Education		−0.2117^*^
		(0.1168)
Marriage		−0.4972^**^
		(0.2426)
Smoke		−0.2264
		(0.2157)
Drink		−0.0634
		(0.1737)
gender		−0.5552^**^
		(0.2240)
Lnincome		−0.0752
		(0.0753)
Lngdp		0.0667
		(0.1865)
Lnmgdp		−0.3534
		(0.4620)
AS		0.2982 (0.3739)
Lnmidu		0.0006
		(0.0010)
Lnphysicians		0.0642 (0.0481)
_cons	9.5405^***^	19.9181^**^
	(0.1424)	(7.8721)
Observations	10,134	10,134
*R-squared*	0.053	0.545

Standard errors are in parentheses, *** *p* < 0.01,*** denote significance at the 1% levels, and the numbers in parentheses are robust standard errors clustered by firms.

### 4.3 Heterogeneity test

#### 4.3.1 Region

The implementation of e-commerce policies in rural areas requires a tailored approach. The level of infrastructure development in rural regions of eastern, central, and western China varies significantly due to differences in population distribution and natural environments. In the western rural areas, the population is relatively dispersed with a lower population density. Furthermore, this region is susceptible to natural disasters, contributing to its relatively underdeveloped infrastructure. This underdeveloped infrastructure is ill-suited to accommodate the rapid development of the “Internet +” era, which hinders the growth of e-commerce. Consequently, the implementation of e-commerce policies in rural areas may have heterogeneous impacts on the depressive symptoms among residents due to regional differences. Table 5 delineates the results of the regression analyses conducted on samples of rural older adult individuals in the eastern, central, and western regions. Specifically, the coefficient for the eastern rural older adult is −1.2035, showing statistical significance at the 1% confidence level; the coefficient for the central rural older adult is −1.8829, also showing statistical significance at the 1% confidence level. Conversely, the coefficient for the western rural older adult does not reach statistical significance at the 10% confidence level. This suggests that the development of rural e-commerce has a more pronounced effect on improving the depressive symptoms among rural older adult individuals in the eastern and central regions.

**Table 5:** Regional heterogeneity in the effect of e-commerce development level.

	East (1)	Central (2)	West (3)
DID	−1.2035^***^	−1.8829^***^	−0.7437
	(0.3899)	(0.3884)	(0.6538)
Age	0.4355^*^	−0.2509	−0.5384
	(0.2507)	(0.1643)	(0.3721)
Srh	−0.8085^***^	−0.5290^***^	−0.5591^***^
	(0.1157)	(0.1193)	(0.1526)
Education	−0.3240^*^	−0.1109	−0.1643
	(0.1816)	(0.2056)	(0.2612)
Marriage	−1.2864^***^	0.1650	−0.2385
	(0.4088)	(0.4022)	(0.4921)
Smoke	−0.2785	0.2862	−0.8185^*^
	(0.3618)	(0.3669)	(0.4217)
Drink	−0.0285	0.1621	−0.0820
	(0.3038)	(0.2909)	(0.3314)
Lnincome	−0.0332	−0.0326	−0.0977
	(0.1356)	(0.1174)	(0.1548)
Lngdp	0.3122	−0.3386	0.7615
	(0.3392)	(0.2735)	(0.6520)
Lnmgdp	−0.3561	−1.3951	−0.2926
	(0.9984)	(0.8579)	(1.3201)
AS	0.2620	0.2677	1.9345
	(0.5790)	(0.6929)	(1.2155)
Lnmidu	−0.0036	−0.0008	−0.0079
	(0.0034)	(0.0016)	(0.0065)
Lnphysicians	0.0581	0.0643	0.5262^**^
	(0.0956)	(0.0831)	(0.2231)
_cons	−12.3370	43.9867^***^	46.7020^*^
	(17.2092)	(13.0271)	(25.7104)
Observations	3,738	3,538	2,693
*R-squared*	0.563	0.541	0.529

Standard errors are in parentheses. *** *p* < 0.01, ** *p* < 0.05, and * *p* < 0.1.

#### 4.3.2 Whether living with children or not

In the context of modernization, driven by the need for survival and development, the offspring of rural older adult individuals often opt to migrate to urban areas for work (67). Consequently, the rural older adult often face the dual challenges of aging and empty-nest syndrome, which result in higher risk of depression. This study posits that the impact of the development level of rural e-commerce on the depressive symptoms among rural older adult individuals may exhibit heterogeneity depending on whether they cohabit with their children. The regression results presented in Table 6 indicate that the coefficient for those not living with their children is −2.1135, which is statistically significant at the 1% confidence level. In contrast, the coefficient for those living with their children is −0.5607, which is not statistically significant at the 10% confidence level. This suggests that the development level of rural e-commerce has a more pronounced effect on improving the depressive symptoms among rural older adult individuals who do not live with their children.

**Table 6:** Heterogeneity between living with and without children.

	Living with their children	
	No (1)	Yes (2)
DID	−2.1135^***^	−0.5607
	(0.3174)	(0.3874)
Age	−0.0952	−0.4791^**^
	(0.1674)	(0.2054)
Srh	−0.5931^***^	−0.6213^***^
	(0.0979)	(0.1207)
Education	−0.2519	−0.3187
	(0.1653)	(0.2127)
Marriage	−0.5931^*^	−0.2144
	(0.3270)	(0.4351)
Smoke	−0.2021	−0.2058
	(0.3082)	(0.3635)
Drink	0.0536	−0.1863
	(0.2320)	(0.3115)
Lnincome	−0.1760^*^	0.0339
	(0.1021)	(0.1230)
Lngdp	−0.0451	0.1043
	(0.2514)	(0.3045)
Lnmgdp	−1.1254	0.3645
	(0.6937)	(0.8192)
AS	0.0619	0.4651
	(0.4925)	(0.7060)
Lnmidu	−0.0002	−0.0032
	(0.0014)	(0.0027)
Lnphysicians	0.1553^**^	−0.0084
	(0.0676)	(0.0883)
_cons	29.4397^**^	42.7505^***^
	(11.5454)	(15.2299)
Observations	5,839	3,573
*R-squared*	0.559	0.559

Standard errors are in parentheses. *** *p* < 0.01, ** *p* < 0.05, and * *p* < 0.1.

### 4.4 Robustness test

To test the robustness of the empirical results, this study conducted the following tests.

#### 4.4.1 Parallel trend test

**Figure 2:**
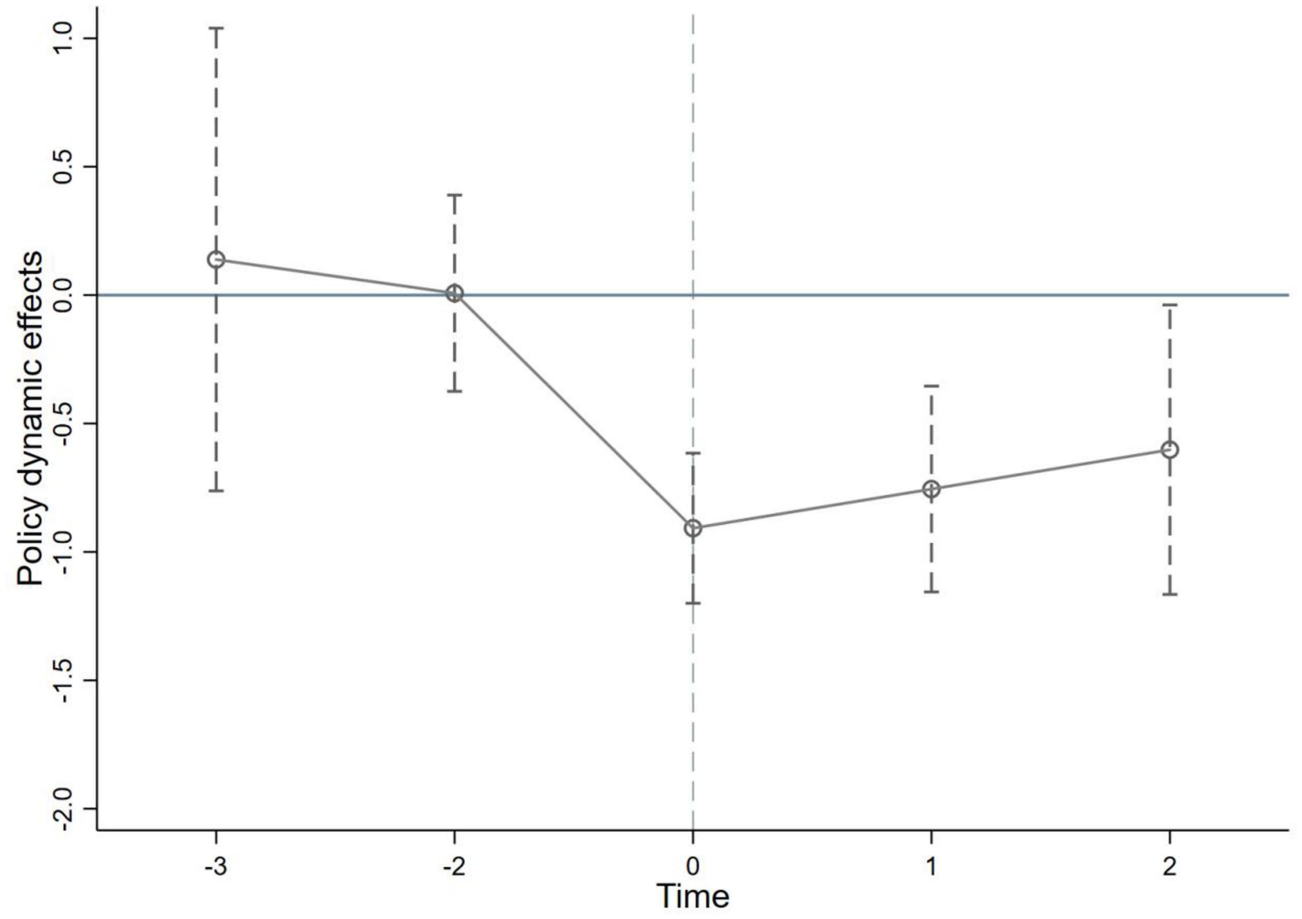
Parallel trends test for rural e-commerce development level.

When using the Difference-in-Differences (DID) method to assess policy effects, the parallel trends assumption must be met. This means that prior to the policy intervention, the core explanatory variables in pilot cities and non-pilot cities should exhibit similar trends. To test this assumption, we use the year of policy implementation as a baseline and examine the effects in the 3 years before and the 2 years after policy implementation. As shown in Figure 2, there is no discernible difference in the pre-policy trend trajectories between pilot and non-pilot cities, thus validating the parallel trends assumption.

#### 4.4.2 Exclude placebo trials with stochastic results

**Figure 3:**
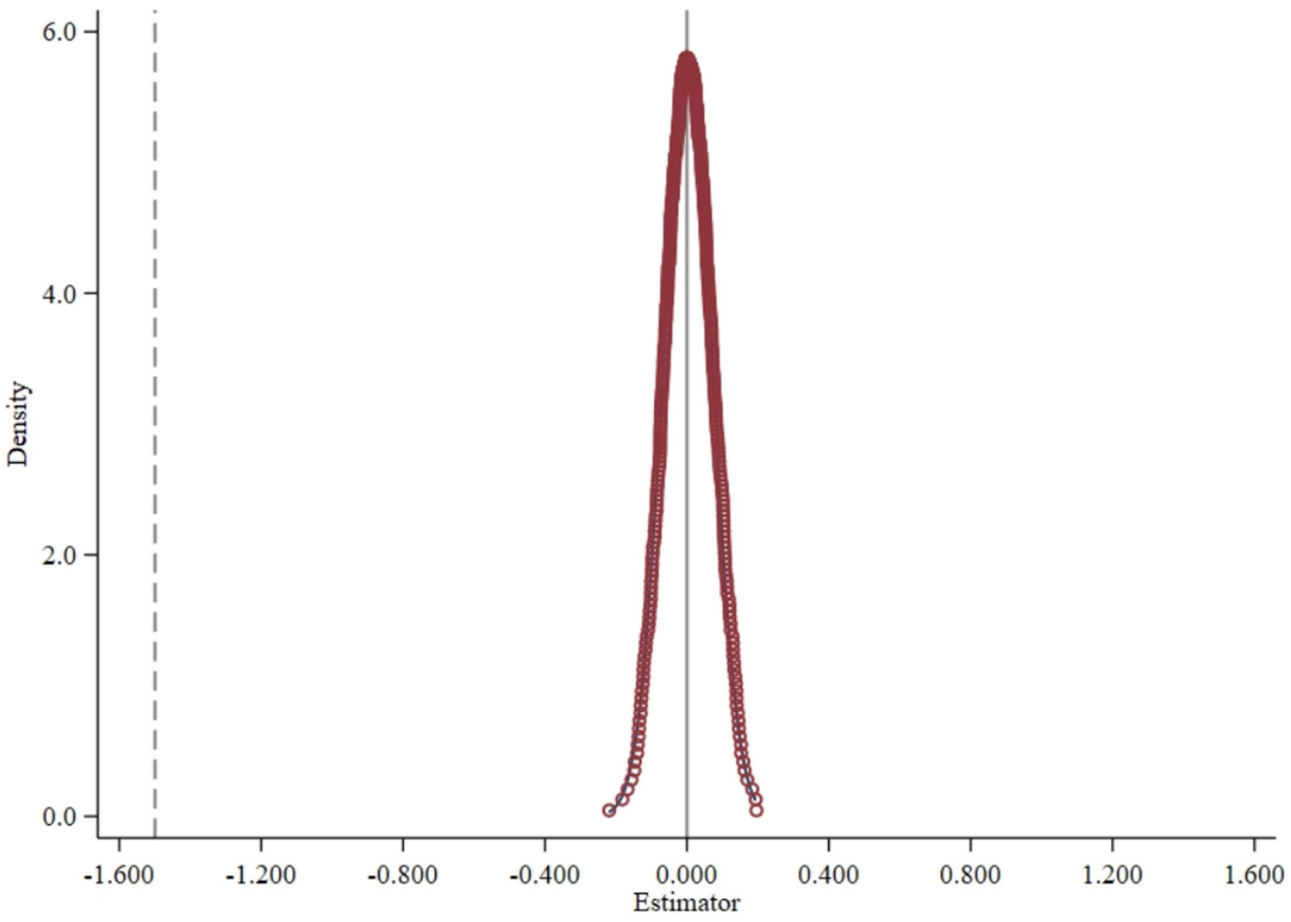
Placebo test chart for e-commerce policy implementation.

To mitigate the influence of stochastic elements, following the approach by Chetty et al. (68), we randomized the years and regions in which the rural e-commerce policy was implemented and repeated this process 500 times in a placebo test. As shown in Figure 3, the distribution of the regression coefficients derived from the randomized simulations is close to zero, while the coefficient from the baseline regression is independent of this distribution. This indicates that the empirical findings observed in this study are not contingent on random or chance factors.

#### 4.4.3 PSM-DID

Most studies typically treat policy exogenous shocks as natural or quasi-natural experiments. However, given that the rural e-commerce policy may be shaped by the investment preferences of e-commerce companies, its implementation is unlikely entirely random (69). This results in the rural e-commerce policy pilot regions not being selected randomly. These factors are included in the error term, leading to a correlation between the explanatory variables and the error term, which introduces self-selection bias. To address this bias, a combined Propensity Score Matching (PSM) and Difference-in-Differences (DID) approach is applied.

First, PSM is employed to match each treated individual from the pilot list of the rural e-commerce policy with the most similar individual from the control group. Then, a one-to-one matching method based on propensity scores is applied (70), excluding unmatched samples. The nearest-neighbor matching mitigates sample self-selection issues, reducing bias in the DID model estimations. The matched data are integrated to create the most comparable treatment and control groups. Finally, a multi-period Difference-in-Differences regression analysis was performed on the integrated data. The results in Table 7 indicate that, even with the inclusion of control variables, the regression coefficients remain negative and are validated at the 1% significance level, further confirming the robustness of the findings.

**Table 7:** PSM-DID Regression results of the effect of e-commerce development level on the depressive symptoms among rural older adult individuals.

	Depression
DID	−1.4446***
	(0.2358)
Age	−0.0925
	(0.1078)
Srh	−0.6506***
	(0.0731)
Education	−0.2106*
	(0.1184)
Marriage	−0.4211*
	(0.2460)
Smoke	−0.1743
	(0.2181)
Drink	−0.0983
	(0.1762)
Lnincome	−0.0866
	(0.0762)
Lngdp	0.2695
	(0.2128)
Lnmgdp	−0.2363
	(0.4669)
AS	0.4296
	(0.3795)
Lnmidu	0.0006
	(0.0010)
Lnphysicians	0.0579 (0.0488)
_cons	20.6760***
	(7.9388)
Observations	9,896
R-squared	0.547

Standard errors are in parentheses. *** *p* < 0.01, ** *p* < 0.05, and * *p* < 0.1.

#### 4.4.4 Endogeneity test

Given the potential presence of unobserved omitted variables and other endogeneity issues, the model may inaccurately estimate the impact of the rural e-commerce policy on the depressive symptoms among rural older adult individuals, possibly leading to an overestimation of the main effect. To address this concern, an instrumental variable (IV) approach is applied to test for endogeneity. A review of authoritative literature on the digital economy and e-commerce reveals that the spatial distance between the region and Hangzhou is commonly used by scholars as an instrumental variable for e-commerce development (71, 72). The reason is that Ant Group, located in Hangzhou and recognized as a leader in e-commerce, establishes Hangzhou as a central hub for e-commerce in China (73). Consequently, regions located farther from Hangzhou are less influenced by the rural e-commerce policy (74). Furthermore, under the exclusivity hypothesis, regional distance serves as a predetermined exogenous geographical factor, unrelated to current levels of older adult depression. Thus, the distance between the older adult individual’s prefecture-level city and Hangzhou is used as the instrumental variable in this study. The endogeneity test results after incorporating the distance variable are presented in Table 8. The distance coefficient is significantly positive, and the Anderson canonical correlation LM statistic is significant at the 1% level, rejecting the null hypothesis of under-identification of the instrumental variable. Furthermore, the Cragg-Donald Wald F-statistic far exceeds the Stock-Yogo critical value for weak instruments at the 10% significance level (16.38). Thus, both the under-identification and weak instrument tests are passed, confirming the appropriateness of using distance as the instrumental variable. Notably, Column (2) indicates that the DID coefficient remains significantly negative, suggesting that after controlling for endogeneity, the rural e-commerce policy effectively improves the depressive symptoms among rural older adult individuals.

**Table 8:** Endogeneity test results with instrumental variables.

	(1)	(2)
	DID	Depression
*Distance*	−0.0144^***^	
	(0.0012)	
DID		−3.9958^***^
		(1.0282)
*LM statistic*	136.832^***^	
*F statistic*	147.577	
*N*	10,134	

Standard errors in parentheses. * *p* < 0.10, ** *p* < 0.05, *** *p* < 0.01.

### 4.5 Mediating effect

The benchmark regression results show that the development of rural e-commerce can improve the depressive symptoms among rural older adult individuals. To further explore the underlying mechanisms, an empirical analysis is conducted to test the proposed mediation effects, specifically Hypotheses 2 and 3. Hypothesis 2 suggests that the development of e-commerce impacts depressive symptoms among rural older adult individuals by increasing social interaction frequency. Hypothesis 3 proposes that e-commerce development influences depressive symptoms by enhancing the financial support by children.

#### 4.5.1 Social interaction frequency among rural older adult individuals

Social interaction is defined as engaging with others in formal or informal settings to maintain social relationships (75), forming the core of social participation. Social relationships established through such interactions are shown to effectively improve the depressive symptoms among older adult individuals (76). Based on this, the social interaction frequency among rural older adult is selected as a mediating variable to examine the impact of e-commerce development on depressive symptoms among rural older adult individuals. Column (1) of Table 9 shows that the regression coefficient of rural e-commerce development on the social interaction frequency among rural older adult individuals is significantly positive at the 1% level, indicating that rural e-commerce development increases the social interaction frequency among rural older adult individuals. Column (2) presents the effect of rural e-commerce development on depressive symptoms among rural older adult individuals after including the social interaction frequency as a mediating variable. The results show that the regression coefficient of rural e-commerce development remains significantly negative at the 1% level, changing from −1.4995 to −0.5718, which is consistent with the logic of mediation effects. The *p*-value for the corresponding Sobel test is 0.0003, significant at the 1% level. Therefore, the social interaction frequency among the older adult plays a mediating role in the effect of rural e-commerce development on depressive symptoms, supporting Hypothesis 2.

**Table 9:** Mediating effects of social interaction frequency.

	(1)	(2)
	Social interaction frequency	Depression
DID	3.7691^***^	−0.5718^***^
	(0.3190)	(0.1262)
Social Interaction Frequency		−0.0148^***^ (0.0041)
Age	−0.0152	−0.0083
	(0.0250)	(0.0098)
Srh	−0.0060	−1.2918^***^
	(0.1429)	(0.0561)
Education	0.5453^***^	−0.4663^***^
	(0.1661)	(0.0653)
Marriage	0.0091	−0.9975^***^
	(0.3992)	(0.1568)
Smoke	0.2913	−0.4231^***^
	(0.3313)	(0.1301)
Drink	−0.3261	−0.1712
	(0.3138)	(0.1232)
Lnincome	1.2088^***^	0.0600
	(0.1625)	(0.0640)
Lngdp	2.4354^***^	−0.2658^*^
	(0.3476)	(0.1369)
Lnmgdp	2.2582^***^	−0.6807^***^
	(0.3767)	(0.1482)
AS	−0.0552	0.3309^**^
	(0.3478)	(0.1366)
Lnmidu	0.0013^**^	−0.0013^***^
	(0.0006)	(0.0002)
Lnphysicians	−0.4821^***^	0.0090
	(0.0459)	(0.0181)
_cons	−24.2247^***^	23.3417^***^
	(3.8890)	(1.5303)
*p*-value	0.0003	

Standard errors in parentheses. ^*^ *p* < 0.10, ^**^ *p* < 0.05, ^***^ *p* < 0.01.

#### 4.5.2 Financial support by children

Advancements in rural e-commerce facilitate children’s financial assistance to their parents, predominantly through the procurement of daily essentials via online platforms. Column (1) of Table 10 shows that the regression coefficient of rural e-commerce development is significantly positive, indicating that rural e-commerce development significantly increases the financial support by children. Column (2) presents the effect of rural e-commerce development on depressive symptoms among rural older adult individuals after including financial support by children as a mediating variable. The results indicate that the regression coefficient of rural e-commerce development remains statistically significant, changing from −1.4995 to −0.6207, which is consistent with the logic of mediation effects. The *p*-value for the corresponding Sobel test is 0.0008, significant at the 1% level. Therefore, financial support by children plays a mediating role in the effect of rural e-commerce development on depressive symptoms among rural older adult individuals, supporting Hypothesis 3.

**Table 10:** Mediating effects of financial support by children.

	(1)	(2)
	Financial support by children	Depression
DID	1065.4819^***^	−0.6207^***^
	(200.4810)	(0.1282)
Financial support by children		−0.00001^***^ (0.0000)
Age	24.5072	−0.0092
	(15.8458)	(0.0101)
Srh	52.4952	−1.2878^***^
	(89.4377)	(0.0571)
Education	512.5391^***^	−0.4550^***^
	(103.3952)	(0.0661)
Marriage	1261.0209^***^	−0.9584^***^
	(254.5142)	(0.1627)
Smoke	−370.3094^*^	−0.4397^***^
	(207.3808)	(0.1324)
Drink	578.6138^***^	−0.1442
	(196.2533)	(0.1254)
Lnincome	401.8832^***^	0.0662
	(101.2551)	(0.0647)
Lngdp	195.8174	−0.2336^*^
	(217.0549)	(0.1386)
Lnmgdp	603.3633^**^	−0.7222^***^
	(235.5935)	(0.1505)
AS	592.2694^***^	0.3623^***^
	(218.4556)	(0.1395)
Lnmidu	1.4426^***^	−0.0013^***^
	(0.3604)	(0.0002)
Lnphysicians	−44.2054	0.0157
	(28.6634)	(0.0183)
_cons	−8261.5076^***^	23.6654^***^
	(2436.5253)	(1.5568)
*p*-value	0.0008	

Standard errors in parentheses. * *p* < 0.10, ** *p* < 0.05, *** *p* < 0.01.

## 5 Discussion

### 5.1 Rural e-commerce can improve the depressive symptoms among rural older adult individuals

The benchmark regression results demonstrate that the development of e-commerce in rural areas is capable of improving the depressive symptoms among rural older adult individuals. Our investigation findings further validate the assertion of Huang et al. that e-commerce platforms can enhance the well-being of rural residents (77). Nevertheless, the distinction lies in that our research focuses on the depressive symptoms among rural older adult individuals.

The potential explanatory mechanisms for this finding are as follows. First, e-commerce policies significantly influence the shopping behaviors of rural older adult individuals. The implementation of the rural e-commerce policy actively promotes the development of rural e-commerce infrastructure, such as expanding internet coverage and optimizing logistics networks. This provides older adult individuals with a convenient online shopping environment, greatly reducing the time and physical effort required for shopping (78). Even those in remote areas can access essential goods and medical supplies via the internet. These improvements enhances the quality of life for rural older adult individuals, increase their sense of autonomy and fulfillment, and, in turn, reduce psychological stress associated with shopping challenges (79). Second, e-commerce policies promote the well-being of rural older adult individuals by enhancing social interaction. E-commerce platforms inherently offer social features, such as product reviews and the sharing of shopping experiences, which facilitate interactions between rural older adult individuals and other users (50). These online virtual interactions not only help alleviate loneliness in the daily lives of rural older adult individuals but also foster a sense of social participation and belonging, thereby improving their mental health (51). Additionally, the increased internet accessibility and improved digital literacy among rural older adult individuals—facilitated by e-commerce policies—likely play a crucial role in further enhancing their mental well-being. As internet coverage expands, rural older adult individuals gain broader access to emotional support and social participation through various online channels, overcoming geographical barriers and effectively alleviating depressive symptoms (80). Furthermore, the widespread adoption of e-commerce platforms also facilitates interaction between older adult individuals and their children. Many children purchase daily necessities for their parents through e-commerce platforms, establishing a new mode of intergenerational interaction (81). This mode not only strengthens the emotional bond between the older adult and their children but also allows the older adult to feel familial support and care, thereby enhancing their mental well-being.

However, the development of e-commerce also introduces potential negative impacts. First, the digital divide remains a significant issue. Older adult individuals with higher digital literacy can more easily navigate e-commerce platforms, interact with others, and communicate, thereby significantly reducing their psychological stress and feelings of loneliness. However, some older adult individuals, lacking digital skills, are unable to fully utilize e-commerce platforms, preventing them from benefiting from the convenience brought by e-commerce (82). This limitation not only heightens their sense of disconnection from society but may also increase feelings of helplessness and isolation. Second, some older adult individuals may become overly reliant on e-commerce, engaging in online shopping excessively (83), particularly when unsupervised by family members. This overreliance may lead to challenges in financial management and even increase financial strain. Furthermore, while the virtual interactions facilitated by e-commerce provide more social opportunities, they may also reduce real-life social engagement to some extent. For older adult individuals who become overly dependent on online interactions, this substitution of virtual socialization may have negative consequences for their depressive symptoms (50).

The positive impacts observed in this study may partly result the unique characteristics of rural e-commerce development. First, the needs of rural older adult individuals tend to be more basic and practical (84), and the convenience of e-commerce platforms meets these needs without the unnecessary consumption that urban older adult individuals may be more prone to. Second, the development of rural e-commerce not only strengthens the economic and emotional ties between older adult individuals and their children but also compensates for the limited physical commercial infrastructure in rural areas. Particularly in remote regions, e-commerce has become the primary channel through which older adult individuals access goods (85). This convenient and efficient shopping method enhances the autonomy of older adult individuals in their daily lives while also providing greater support for their mental well-being. Finally, the social effects of e-commerce are particularly evident in rural areas. Compared to urban regions, rural areas have lower population density and fewer commercial activities, leading to narrower social networks and more limited social opportunities (86). Unlike general social media or the internet, e-commerce promotes dual interaction—with both society and family—by meeting the practical needs of older adult individuals. This dual function offers unique advantages in alleviating the depressive symptoms among rural older adult individuals, and provides effective new approaches for addressing the depressive symptoms among rural older adult individuals.

Although this study focuses on the impact of e-commerce on depressive symptoms among rural older adult individuals, its effects are often closely related to the widespread adoption of the internet and the enhancement of digital literacy among seniors. The internet’s coverage and the improvement of older adult individuals’ digital skills not only facilitate the use of e-commerce but also provide broader online social opportunities, collectively contributing to improved mental health.

### 5.2 The heterogeneous effects

#### 5.2.1 Heterogeneous effects of region

The heterogeneity effect analysis in Table 5 reveals that the impact of e-commerce development on depressive symptoms among rural older adult individuals is more pronounced in the eastern and central regions. First, in terms of infrastructure, the eastern and central regions possess relatively advanced logistics and communication networks (87), allowing for a more established presence of e-commerce. Older adult individuals in the eastern region, in particular, have greater access to the conveniences of e-commerce, which contributes to improved quality of life and depressive symptoms. Geographical constraints in the western region result in relatively underdeveloped infrastructure, limited internet coverage, and incomplete logistics services, leading to a lower level of e-commerce development (88). Consequently, opportunities for older adult individuals to access and use e-commerce are limited, resulting in less pronounced improvements in depressive symptoms compared to the eastern and central regions. Additionally, regional economic disparities affect the acceptance of e-commerce among the older adult. The relatively developed economies of the eastern and central regions result in higher household income levels for rural older adult individuals, enabling greater engagement with e-commerce and the purchase of both essential and non-essential goods (89). In contrast, in the western region, particularly in remote rural areas, lower income levels among the older adult limit their e-commerce use primarily to essential daily goods (90). This restriction in purchasing power contributes to a relatively lower sense of psychological satisfaction derived from e-commerce engagement. Additionally, cultural and digital literacy differences affect older adult individuals’ willingness and ability to use e-commerce. In the eastern and central regions, higher educational levels and ongoing digitalization enable many older adult individuals to gradually acquire skills in using smartphones and the internet (91). This proficiency facilitates the integration of older adult into the digital society, enabling them to benefit from the conveniences of e-commerce. However, in the western region, particularly in remote areas, older adult individuals often have lower educational attainment and lack digital skills, making e-commerce use challenging (92). This digital divide not only restricts their access to e-commerce opportunities but also exacerbates social isolation, further impacting their depressive symptoms (93). Finally, differences in social support systems contribute significantly to regional heterogeneity. In the eastern and central regions, governments and communities have established well-developed training programs and digital operational systems to help older adult individuals adapt more effectively to the growth of e-commerce (94). In contrast, in the western region, particularly in economically disadvantaged rural areas, guidance and promotion of e-commerce normalization are limited (95), Age-friendly services and technical support are insufficient, making it challenging for older adult individuals to benefit from the depressive symptoms improvements associated with e-commerce participation.

#### 5.2.2 Heterogeneous effects of whether live with children or not

The analysis presented in Table 6 delineates a more pronounced effect of the development of rural e-commerce on the depressive symptoms among rural older adult individuals who do not cohabitate with their children. This phenomenon is likely attributable to the reduced availability of familial care and financial support for older adult individuals without their children living nearby, which may exacerbate feelings of solitude. Therefore, these individuals are more dependent on broader social support systems to sustain their mental well-being. The emergence of e-commerce promotes the communication between the rural older adult and their children, increases the children’s financial support for their parents, and can help them reduce their loneliness and improve their depressive symptoms.

## 6 Conclusions and policy implication

### 6.1 Conclusion

Drawing upon the multi-period data from the CHARLS survey, this study examines the influence of rural e-commerce development on the depressive symptoms among rural older adult individuals, leading to the following conclusions: (1) The advancement of rural e-commerce has positively impacted the depressive symptoms among rural older adult individuals, with the findings maintaining statistical significance across parallel trend, placebo, Endogeneity, and PSM-DID tests. (2) Heterogeneity analysis reveals a more substantial effect of e-commerce development on the depressive symptoms among older adult individuals in eastern and central regions, as well as those not living with their offspring. (3) Mechanistically, rural e-commerce development influences depressive symptoms by boosting social interaction frequency among rural older adult individuals and enhancing the financial support by children. These findings offer empirical support for further inquiry into rural e-commerce development and for initiatives aimed at improving the depressive symptoms among rural older adult individuals.

### 6.2 Policy implication

This study finds that the rural e-commerce policy has a positive effect on alleviating depressive symptoms among rural older adult individuals. Future policies should address the specific needs of different regions and groups through targeted measures to further expand e-commerce coverage and enhance its impact, thereby maximizing its benefits for depressive symptoms among rural older adult individuals.

1. Promote the development of age-friendly e-commerce platforms to improve digital skills among the older adult. E-commerce provides rural older adult individuals with both convenience in daily life and new social opportunities. However, older adult individuals with limited digital skills, particularly in the western region, often struggle to fully benefit from these platforms. Therefore, national efforts should focus on developing age-friendly e-commerce platforms that simplify navigation and incorporate features such as voice assistance, making them more accessible to the older adult. Additionally, local governments should enhance digital skills training for older adult individuals by organizing regular, age-appropriate technology workshops. Such initiatives help older adult individuals acquire essential skills in operating smart devices and using the internet, thereby bridging the digital divide and ensuring that more older adult individuals benefit from the conveniences and depressive symptoms improvements associated with e-commerce.
2. Strengthen differentiated support across eastern, central, and western regions to compensate regional development disparities. The rapid development of e-commerce in the eastern and central regions has led to significant improvements in depressive symptoms, while limited infrastructure in the western region constrains e-commerce coverage and usage. Therefore, policymakers should adopt differentiated support strategies based on regional disparities. For the western region, increased government investment in infrastructure is necessary to enhance internet coverage and logistics efficiency, ensuring that rural older adult individuals have easy access to e-commerce^112^. Additionally, in promoting e-commerce platforms, local governments should integrate resources to encourage returning entrepreneurs and recent college graduates to develop e-commerce businesses locally. This approach supports both rural economic growth and improvements on the depressive symptoms among older adult individuals.
3. Enhance family and community support systems to encourage intergenerational interaction. This study demonstrates that rural older adult individuals who do not reside with their children derive greater benefits from e-commerce, suggesting that e-commerce may partially compensate for the lack of family support in this population, further reinforcing the findings of Topino et al. (96). Accordingly, policies should encourage children to provide financial support and foster emotional interaction with their parents via e-commerce platforms. For example, e-commerce platforms could improve and expand features such as “family payment” and “proxy purchasing,” making it easier for children to purchase essential items for parents living in rural areas. Additionally, fostering interactions between older adult individuals, their children, and neighbors through community activities and volunteer services helps to bridge the emotional gap caused by generational separation, thereby further improving depressive symptoms among rural older adult individuals.
4. Improve e-commerce oversight and protection mechanisms to prevent excessive dependence among the older adult. Although e-commerce provides convenience for the older adult, some individuals may develop over-reliance and even face financial management challenges. To mitigate these negative effects, comprehensive regulatory mechanisms should be established by the government and e-commerce platforms, including features such as purchase reminders and spending management to help older adult users plan online purchases responsibly and avoid overspending. Additionally, communities should strengthen consumer education for the older adult, helping them identify fraudulent advertisements and scams to ensure safe e-commerce experiences.

### 6.3 Limitations

First, despite the control of multiple variables related to the demographic characteristics and social environment of rural older adult individuals in this study, these variables may not fully encompass all fundamental confounding factors influencing the depressive symptoms among rural older adult individuals. Second, the depressive symptoms data of rural older adult individuals are derived from self-reports, which may not accurately reflect their actual conditions. Third, due to data availability constraints, the CHARLS database does not provide information on actual e-commerce usage by rural older adult individuals, such as usage frequency or spending amounts. Consequently, this study examines the impact of e-commerce development on depressive symptoms among rural older adult individuals primarily from the perspective of e-commerce development levels. Future research could expand data sources to facilitate more in-depth analysis. Fourth, although we introduced instrumental variables to help mitigate endogeneity issues and used the PSM-DID model to address sample self-selection, we acknowledge that other factors may still contribute to potential endogeneity and omitted variable bias. Fifth, the heterogeneity analysis in this paper is limited to the three main economic regions of China: eastern, central, and western. Future research could delve into a more nuanced exploration of the effects of policy implementation on the depressive symptoms among rural older adult across various city sizes and cultural backgrounds.

## Data Availability

The original contributions presented in the study are included in the article/supplementary material, further inquiries can be directed to the corresponding author.
